# Comprehensive Therapy for Infant Vascular Tumor Associated With Kasabach–Merritt Phenomenon—Single-Center Primary Experience

**DOI:** 10.3389/fped.2022.924422

**Published:** 2022-06-22

**Authors:** Xiaoting Sun, Miao Xu, Kaiyang Lv, Xiaorong Ma, Liming Wu, Tianxiang Ouyang

**Affiliations:** ^1^Department of Plastic and Reconstructive Surgery, Xinhua Hospital, Shanghai JiaoTong University School of Medicine, Shanghai, China; ^2^Department of Endocrinology, Xuhui District Central Hospital, Shanghai, China

**Keywords:** Kasabach Merritt phenomenon, kaposiform hemangioendothelioma, tufted angioma, intralesional photocoagulation, sclerotherapy

## Abstract

**Objective:**

To introduce our single-center experience of infant vascular tumor associated with Kasabach–Merritt phenomenon (KMP) which received combined medicine treatment with intralesional laser photocoagulation (ILP) and sclerotherapy.

**Methods:**

A retrospective study was conducted using medical records of all children with a diagnosis of kaposiform hemangioendothelioma (KHE) or tufted angioma (TA) associated with KMP treated with medicine, intralesional laser photocoagulation (ILP), and sclerotherapy between February 2017 and November 2020. Clinical features, response to comprehensive therapy, and outcomes were recorded.

**Results:**

A total of 23 patients including nine females (39%) and 14 males (61%) were identified. The mean age was 6.9 months (age range, 11 days−2 years) at the time of treatment. Nine children (39%) demonstrated sensitivity to single corticosteroid therapy; 14 children (61%) received combined therapy with intravenous Vincristine (VCR) and corticosteroid therapy. All children had at least two ILP and sclerotherapy performed, with a mean of 3.5 procedures (range: 2–6). Of these 14 children, only one experienced a relapse of thrombocytopenia and the remaining 13 children had no clinical symptoms recurred.

**Conclusion:**

The combined therapy modalities could induce a more rapid tumor response and resolution of KMP and decrease the rebound rates. This research presents a novel and safe multi-modality treatment for infant vascular tumors associated with KMP.

## Introduction

The Kasabach–Merritt phenomenon (KMP) is a condition that causes thrombocytopenia, microangiopathic hemolytic anemia, and consumption coagulopathy within a vascular tumor, either kaposiform hemangioendothelioma (KHE) or tufted angioma (TA). KHE is more frequently complicated by KMP than TA. KMP was first reported by Kasabach, a radiologist, and Merritt, a pediatrician in 1940. Although KMP is rare, affecting <1% of all children with vascular tumors, it can be life-threatening because of the involvement of multiple organs and massive bleeding. About 80% of patients present within 1 year after birth, and the reported mortality rate ranges from 10 to 37% (1). Severe complications and high mortality urge aggressive intervention.

Various approaches have been clinically used to treat KMP, including medicine, surgeries, intravascular embolization, and radiation therapy. Due to its rarity, there exists no strong evidence basis behind many of the treatment options for KMP. Clinicians rely on case reports, limited retrospective series, and expert opinions when selecting therapeutic interventions for complicated patients. Although consensus-derived treatment protocols for the first-line treatment have been issued in 2013 (2), the management of complicated and refractory cases is still difficult. In the present study, we report our single-center experience of infant vascular tumor associated with KMP which received combined medicine treatment with intralesional laser photocoagulation (ILP) and sclerotherapy.

## Patient and Methods

### Clinical Data

The retrospective chart review was performed on 23 patients who suffered from KMP between February 2017 and November 2020 at the Xinhua Hospital affiliated with Shanghai Jiaotong University School of Medicine. The Ethics Committee of Xinhua Hospital approved this study. The diagnosis of KMP was made by a hematologist and a plastic surgeon, defined as KHE or TA with thrombocytopenia, microangiopathic hemolytic anemia, and consumption coagulopathy. Magnetic resonance imaging was used to confirm the characteristics and range of the lesion.

Demographic data including patient age, gender, medications, and details of ILP and sclerotherapy were recorded. The hospital records were reviewed. The clinical presentation and physical findings were recorded in all cases. Bleeding symptoms included bruising, petechiae, melena, hematemesis, or overt bleeding. Laboratory data at presentation, during and post-treatment, were collected. Hematologic data included complete blood count, prothrombin time, activated partial thromboplastin time, fibrinogen, and D-dimers. The modalities of treatment, along with the response to therapy and outcome were documented.

Response to treatment was defined as a recovery in hemostatic parameters, with or without a reduction in the size of the vascular tumor. Radiological imaging was used to document a reduction in the size of the tumor by objective measurement.

### Systemic Corticosteroids and VCR Treatment

Patients received oral corticosteroids as first-line treatment (prednisone, 4–5 mg/kg of bodyweight per day). After observation for the initial pharmacologic therapy, the patients were discharged and returned for weekly blood tests to evaluate the curative effect. When the platelet count normalized nearly >80 × 10^9^/L, the patients were readmitted for ILP and sclerotherapy. For the steroid-resistant case whose platelet counts did not increase significantly (>20 × 10^9^/L) within 2 weeks, intravenous VCR was added until the recovery of the thrombocytopenia.

### ILP and Sclerotherapy

To achieve a more rapid resolution of KMP, ILP and sclerotherapy were carried out. Under general anesthesia, a core needle biopsy was attempted for the pathologic diagnosis if informed parental consent was obtained for invasive procedures. We applied ILP to the lesion at a power of 6–10 W and in the continuous mode using a Diode laser system at a 980-nm wavelength (LASEmaR1000; EUFOTON S.R.L, Italy) with a 600 μm fiber. The laser fiber was inserted into the vascular lesion through perforations on normal skin near the lesion. Irradiation was delivered through the fiber. To fulfill the laser to the entire lesion, multiple perforations and multiple directions of laser tunnels were made. Several skin perforations were needed to access the entire lesion. To avoid an unexpected blister on the lesion surface, ice gauze was used to cool down the surface temperature of the lesion.

After ILP, multiple site percutaneous injections around the original tumor were performed by using 24G “butterfly” needles connected with a syringe. After confirming entrance into the vascular lumen by retrograde blood flow, 0.2–0.3 ml of absolute ethanol was injected slowly into each site with a total volume of <0.5 ml/kg. When a few clotting particles appeared in the retrograde blood flow, the needle was retained and the connecting syringe was changed to another one, which contained a mixture of 5 ml (50 mg) polyoxyethylene lauryl ether (Lauromacrogol, Tianyu Corp., China) and 1 ml (7 mg) compound betamethasone (Diprospan, Schering-Plough Corp., United States) with 5 mg methotrexate (Pude pharma, Shanxi, China). Appropriate volumes of the mixture (<0.5 ml) were injected slowly with repeated aspiration in the same site until more clotting particles appeared in the retrograde blood flow. After removing the needle and hemostasia by slight compression, the same procedure was performed in the adjacent site at 2–3 cm intervals.

Before the sclerotherapy, if the original tumor was located in the limbs, an attempt was made to reduce the blood flow back to the heart by tourniquet to reduce systemic complications of the sclerotherapy including cardiac arrest and pulmonary embolism. The tourniquet was then gradually removed to avoid sudden systemic release of the sclerosant.

Clinical observation including coagulation parameters was evaluated every other day for 1 week. Then, the patient was discharged, and attendance of regular follow-up visits was requested to assess the need for further IPL and sclerotherapy. Successive procedures were performed in a minimum of 4–8 weeks apart, on the patients with the recurrence of symptoms or non-involuting tumor. Systemic corticosteroid therapy was performed concurrently during the first sclerotherapy and then was slowly tapered through 1 month.

## Results

### Patients

Between February 2017 and November 2020, 23 patients (14 males, 9 females) with KMP were identified. The demographic data, clinical features, and treatment outcomes of these patients are summarized in Table 1. There was a slight male pre-ponderance, and the median age at presentation was 7 days (range: 1 day−24 months). All children presenting to our hospital had a large mass combined with a decreased platelet count. All the children had a single cutaneous lesion, six of which involved multiple regions. Cutaneous lesions favored the extremities (15/23), especially overlying joints and functional impairment. The mean age was 6.9 months (age range, 11 days−2 years) at the time of treatment.

**Table 1:** Clinical data and results of 23 patients.

**Patient No**	**Gender**	**Age at treatment (month)**	**Location of lesion**	**Methyl-** **prednisolone**	**Vincristine**	**Platelet count,10** ^**9**^ **/L**		**No of IPL + sclerotherapy**	**Complication**
						**Before treatment**	**After treatment**		
1	M	4	Submandiblar region, neck, and chest wall	Yes	Yes	13	388	6	Cushing appearance
2	M	4	Right upper arm	Yes	No	15	217	4	Skin blister after IPL, pigmentation
3	F	4	Left back	Yes	Yes	12	276	3	Pigmentation
4	M	11 days	Left thigh	Yes	No	14	245	3	Cushing appearance
5	F	16	Right knee	Yes	No	43	310	2	Skin blister after IPL
6	F	2	Right arm	Yes	Yes	7	411	4	Pigmentation
7	M	3	Left face and neck	Yes	No	27	367	5	Skin blister after IPL, pigmentation
8	F	3	Left foot	Yes	No	47	490	2	Pigmentation
9	F	2	Right thigh	Yes	No	23	280	4	Cushing appearance
10	M	7	Left foot	Yes	Yes	18	371	4	No
11	M	3	Right knee and thigh	Yes	Yes	9	316	3	Pigmentation
12	F	8	Back	Yes	Yes	18	410	5	No
13	M	4	Right upper arm	Yes	Yes	15	254	3	Skin blister after IPL, pigmentation
14	M	14	Right hip	Yes	No	41	306	2	Pigmentation
15	F	5	Left foot	Yes	Yes	13	298	4	No
16	M	3	Left leg	Yes	Yes	14	216	4	Pigmentation
17	M	4	Right foot	Yes	Yes	17	283	2	Pigmentation
18	F	15	Right arm	Yes	Yes	23	341	3	No
19	M	12	Chest wall	Yes	No	41	291	4	Cushing appearance
20	M	7	Left shoulder	Yes	Yes	12	303	5	Pigmentation
21	F	8	Neck, chest wall	Yes	Yes	18	318	2	Cushing appearance
22	M	24	Waist	Yes	No	80	248	3	Pigmentation
23	M	6	Left arm	Yes	Yes	24	327	4	No

### Hematological Findings

At presentation, the mean platelet count was 23 × 10^9^/L (range: 7–80 × 10^9^/L) while the mean fibrinogen level was 1.1 g/L (range: 0.6–1.45). Most patients (18/23) were anemic, with mean hemoglobin of 78.5 g/L (range: 49–145).

### Treatment and Response

A total of nine children (39%) demonstrated sensitivity to single corticosteroid therapy with significant improvement in platelet and fibrinogen within 2 weeks. After a mean of 5.5 weeks (range: 4–7 weeks) of corticosteroid therapy, the platelet count was nearly normalized (>80 × 10^9^/L). A total of 14 children (61%) received combined therapy with intravenous VCR and corticosteroid therapy. After a mean of 3.8 weeks (range: 2–5 weeks) of combined therapy, the platelet count was nearly normalized (>80 × 10^9^/L).

Before ILP and sclerotherapy, the mean platelet count was improved to 285 × 10^9^/L (range: 81–450) by pharmacologic therapy. In total, all children had at least two ILP and sclerotherapy performed, with a mean of 3.5 procedures (range: 2–6). Of those, 56.5% (range: 1–4) were performed on the first admission. One week after the last procedure in the first admission, the mean platelet count increased to 285 × 10^9^/L (range: 167–412). Meanwhile, the consumption coagulopathy also gradually recovered. The final results of the treatment of KMP are shown in Table 1. A 9–64 month follow-up evaluation was obtained for all treated children, and 14 children required readmissions for subsequent sclerotherapy aiming to promote tumor regression. Of these 14 children, only one experienced a relapse of thrombocytopenia and the remaining 13 children had no recurrence of clinical symptoms. After repeated pharmacologic therapy and four procedures of ILP and sclerotherapy, the recurring thrombocytopenia recovered finally with tumor regression.

### Complications

The most common complication of ILP and sclerotherapy was localized skin blister and pigmentation. No severe adverse reactions were noted during the treatment courses, such as distal embolization, local tissue necrosis, or thinning of the skin.

### Case Presentation

#### Patient 1

One week after the cesarean section, a full-term female newborn developed red patches on her submandibular region. The tumor progressed and resulted in the involvement of the lower face, neck, and chest wall. Clinical-laboratory findings indicated no consumptive coagulopathy in the first 2 months. The patient was also initially diagnosed with infantile hemangioma and received urea embolization. The lesion still progressed after urea embolization and the patient suffered labored breathing. At the age of 3 months, the patient visited our hospital and the platelet count was 13 × 10^9^/L. Magnetic resonance imaging (MRI) demonstrated a vascular lesion extending from the lower face to the chest wall. The imaging-study findings and platelet consumption permitted the diagnosis of KHE with KMP.

Gamma globulin was given at ages 98 and 99 days. Methylprednisolone was transfused at age 98 days (starting dose 10 mg/kg/day for 3 days; thereafter, slowly tapering to 5 mg/kg/day for 3 days, and 3 mg/kg/day for 3 days). Oral methylprednisolone (12 mg/day) lasted for 11 weeks. Two doses of vincristine were given at ages 98 days (vincristine 1 mg/m^2^) and 105 days (vincristine 0.5 mg/m^2^). Two days after treatment (at age 100 days), the platelet count rose to 102 × 10^9^/L and gradually to 420 × 10^9^/L at age 106 days.

**Figure 1:**
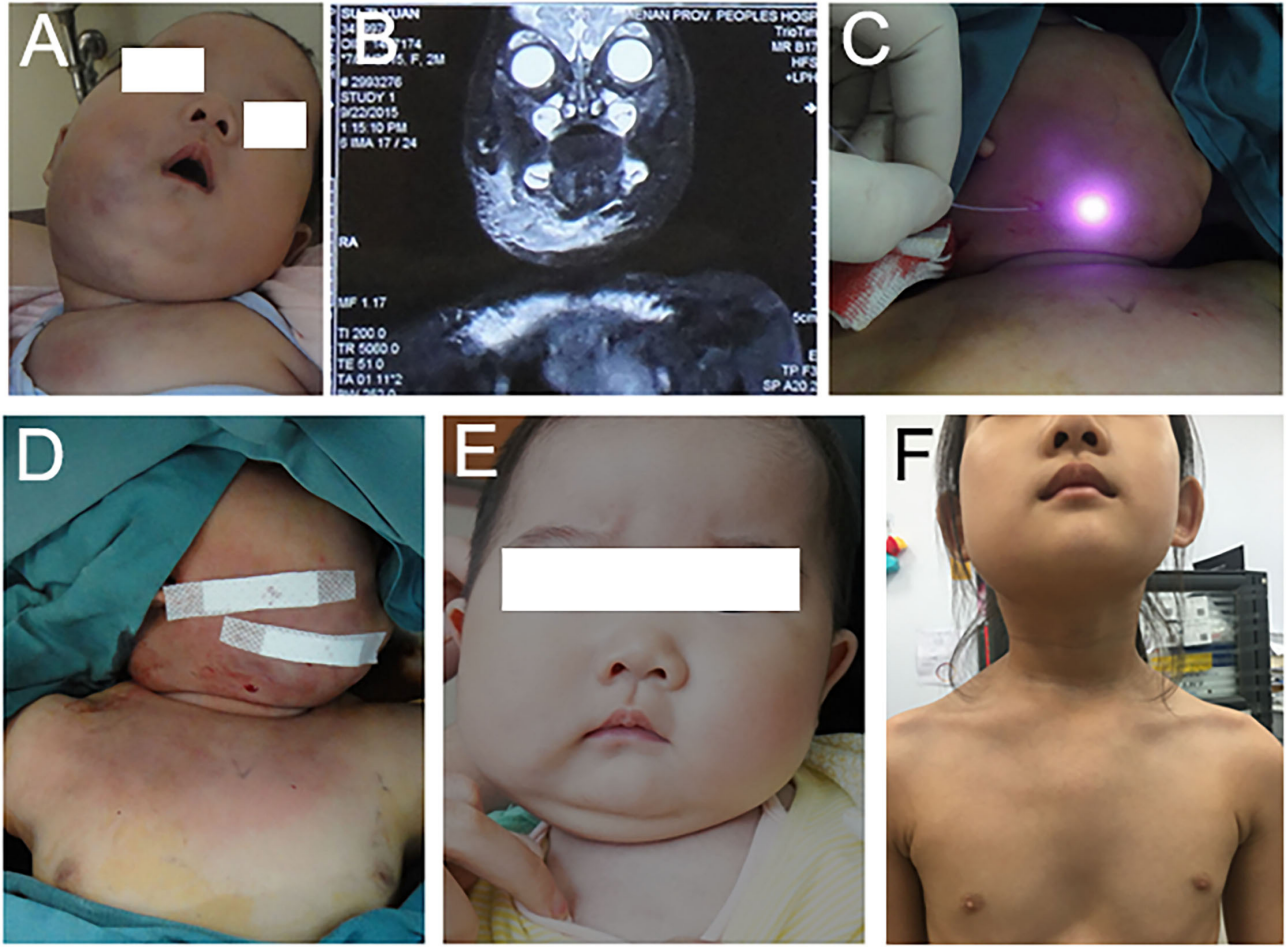
The patient was diagnosed with KHE with KMP. **(A)** The patient showed a lesion on her right face, chin neck, and chest wall at 2 months. **(B)** Magnetic resonance imaging with contrast enhancement performed at 2 months. **(C,D)** The patient received the first course of IPL and sclerotherapy at 4 months. **(E)** The tumor recessed at 10 months. **(F)** The tumor recessed at 6 years.

At the age of 114 days, the patient received ILP and sclerotherapy while the platelet count was normal. A total of six courses of ILP and sclerotherapy were carried out in the first 3 years of the patient and the platelet was normal during the whole period. The lesion decreased significantly in size and the KMP resolved without evidence of relapse after 6 years of follow-up (Figure 1).

#### Patient 2

**Figure 2:**
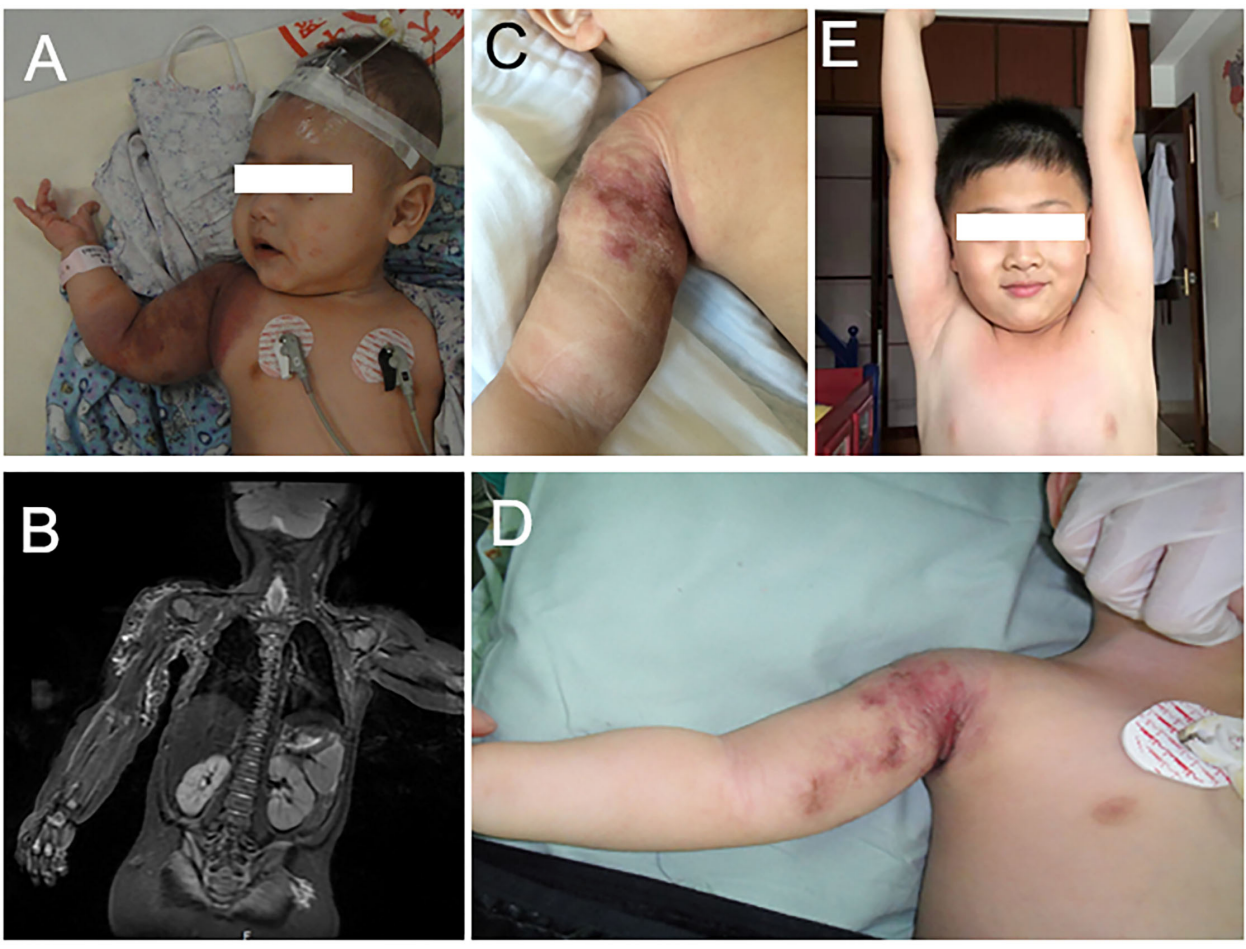
**(A)** The patient was diagnosed with KHE with KMP on his right shoulder and arm at 4 months. **(B)** Magnetic resonance imaging with contrast enhancement revealed the vascular lesion on his right shoulder and arm. **(C)** The lesion recessed obviously after three courses of ILP and sclerotherapy. **(D)** The tumor recessed after three courses of ILP and sclerotherapy. **(E)** The tumor absolutely recessed at 6 years.

A healthy full-term male was noted to have needle-point hemorrhagic spots on his right shoulder and upper arm 2 months after birth. The patient was initially diagnosed with infantile hemangioma and no treatment was initiated. The platelet count showed no thrombocytopenia during the period (124–248 × 10^9^/L). Two weeks before the patient visited our department, the lesion growth resulted in the involvement of the whole upper right arm, shoulder, and scapula region. The patient was treated with propranolol 4.4 mg, three times every day. Despite 2 weeks of therapy, the tumor continued to grow. The platelet count decreased to 47 × 10^9^/L. On the day of visiting our department (at the age of 4 months), the platelet count decreased to 15 × 10^9^/L. Thus, therapy with propranolol was discontinued. Gamma globulin and methylprednisolone were carried out on the first day in the hospital. One unit single-donor platelet and half unit erythrocyte suspension were also transfused. The palate count reached 490 × 10^9^/L on the second morning and decreased to 319 × 10^9^/L on the fifth day. On the seventh day in our hospital, the patient received ILP and sclerotherapy while the platelet count was 362 × 10^9^/L. On 1, 3, and 5 months after the first course of ILP and sclerotherapy, the patient received a second, third, and fourth course of ILP and sclerotherapy. During these 5 months, the platelet was between 183 and 217 × 10^9^/L. The lesion decreased significantly in size and the KMP resolved without evidence of relapse after 6 years of follow-up (Figure 2).

## Discussion

Two types of vascular tumors are associated with KMP, namely KHE and TA. There are other vascular malformations associated with coagulopathy, such as congenital hemangioma, angiofibroma, composite hemangioendothelioma, venous malformations, Kaposiform lymphangiomatosis (KLA), and even the malignancies. However, most of them only manifest as mild coagulopathy. In contrast to true KMP, the abnormal laboratory findings are self-limiting and are usually not complicated by bleeding episodes. Except for an essential close follow-up and recheck, the core needle biopsy also should be attempted for the pathologic diagnosis and the most appropriate treatment if informed parental consent was obtained for invasive procedures. Considering the widespread use of percutaneous sclerotherapy for vascular lesions, a simultaneously performed core needle biopsy is feasible without extrainvasive puncture or anesthesia procedures.

The significant morbidity and mortality associated with KMP necessitate aggressive treatment aiming at the primary vascular tumor, but the management tends to vary, with different responses to the same treatments. Complete surgical resection has been touted as the gold standard for the cure of tumors, but KHE and TA are often unresectable due to their large size and infiltrating nature. Tumor embolization in combination with pharmacologic, surgical, or radiation therapy has been used with a limited degree of success. On reviewing the literature, most first-line treatments of tumors with KMP were curative therapy based, involving systemic corticosteroids, VCR, alpha-interferon (IFN), cyclophosphamide, and more recently, sirolimus (3). Since there are no prospective studies regarding the treatment of KMP or standardized outcome measures to document responses, or systematically obtained long-term follow-up data, the treatment guidelines for KMP have not been established. One of the few consensuses for KMP treatments was achieved in a multicenter and interdisciplinary survey reported by Tlougan in 2013 (4). In this survey, corticosteroid combined with vincristine had the highest degrees of approval in the first-line treatments, and the monotherapy of corticosteroid and vincristine ranked second and third, respectively.

Before the advent of propranolol in infantile hemangioma treatment, corticosteroids were commonly used for the suppression of the vasculogenic potential of hemangioma-derived stem cells (1). In KMP treatment, it appears to increase platelet longevity, increase vasoconstriction, and inhibit fibrinolysis (5). For the possible resistance to corticosteroids, VCR is often regarded as a reliable alternative to control KMP by the apoptosis of endothelial cells and antiangiogenesis (6). In our cohort, these two drugs were selectively combined and did show a positive response with an increase in the platelet count (>20 × 10^9^/L more than the baseline) and fibrinogen (>1.6 g/L) after <4 weeks. The duration of pharmacologic therapy should depend on whether there has been a stabilization of the patient's hematologic status and not on whether there has been an obvious tumor regression. In literature, the intended length of corticosteroid and vincristine therapy was variable ranging between 1 month and 1 year and typically 20–24 weeks, respectively (5, 6). However, it is rare to see complete clearance of this tumor, even with prolonged pharmacologic therapy. Residual tumor or fibrosis is common, especially in more aggressive lesions, and is not a reason to continue therapy if high-risk symptoms have resolved and the tumor is stable on serial imaging (5).

Shapshay first reported ILP for the treatment of vessel anomalies (7). Subsequently, intralesional therapy has been utilized in different kinds of hemangioma and venous malformations, sole or in conjunction with other adjuvant modalities (8, 9). However, no report has been published about ILP in treating infant vascular tumors associated with KMP. Based on our previous treatment success with proliferating infantile hemangiomas and vascular malformation, we treated pediatric KMP patients using ILP and percutaneous sclerotherapy to avoid relapse and promote tumor regression. According to the pathology research of KMP, arteriovenous shunts and the turbulence blood flow that results from the small, convoluted capillaries rising directly from large vessels serially or linearly cause abnormal platelet activation and aggregation (10, 11). By intratumoral laser, laser energy can be delivered directly into deep lesions through a fiber, thereby, effectively coagulating the malformed vessels in the deep lesion. The photocoagulation effect of intratumoral laser can immediately reduce the arteriovenous shunts and turbulence blood flow. KHE and TA cells express podoplanin, which is the only known endogenous ligand for CLEC2 (12). CLEC2 is a C-type lectin receptor that is highly expressed on platelets and is known to elicit powerful platelet activation upon engagement by podoplanin. Photocoagulation destroys the endothelial cells of the lesion and could reduce platelet aggregation and activation.

Afterward swelling of the lesion further results in pressure on the malformed vessels, which can slow down the blood flow in the lesion and make it safer to inject sclerosant. Embolization alone seems to be associated with a high relapse rate of KHE. The sole injection of absolute ethanol or corticosteroid as reported in literature might be diluted easily due to the high flow rates (13, 14). But, Brill (15) reported that transarterial embolization in adjunct to sirolimus treatment provides a more rapid resolution of KMP. Therefore, the sclerotherapy at present was performed in two-step each injection site. In the first step, the absolute ethanol destroyed the downstream vessel including the possible arteriovenous shunts within the tumor. It worked through inducing thrombosis by denaturing blood proteins, denuding the vascular wall of endothelial cells, precipitating their protoplasm, and segmentally fracturing the vascular wall to the level of the internal elastic lamina (16). With the increasing lumen pressure resulting from the obstructed downstream vessel, the originally closed potential vessels were turned on and then were further obstructed by the absolute ethanol in the blood flow. In the second step, Lauromacrogol combined with the compound betamethasone was used to block the remaining vessels and inhibit angiogenesis in the long term.

Intralesional laser photocoagulation is partly a “blind” technique, so there is a risk of unintended destruction of surrounding tissue. It is better to use ultrasound navigation for ILP (9). Nerve damage is a common complication. In the report of Burstein (8), 2 of 100 patients experienced residual weakness of midface branches of the facial nerve after laser treatment of the lesion of the face. Fortunately, no nerve damage occurred in our cases. Cutaneous burns are the other common complication. Since it happens frequently, we paid special attention during the ILP. We kept the fiber at 5–10 mm underneath the surface of the lesion as recommended. Ice cooling further reduced the thermal damage to the skin. No iatrogenic ulceration, perforation, or bleeding occurred in all cases. Potassium may be released from intracellular to extracellular milieu due to hemolysis after ILP and some cytokine or inflammatory factor. ECG, urine test, and blood tests (including whole blood count, potassium concentration, DIC, and renal function) should be carried out immediately after the treatment and the next morning. Intensive care and continued monitoring management collection of information are considered necessary.

The current series represents the first reported experience with a drug in conjunction with ILP and percutaneous sclerotherapy for infant vascular tumors associated with KMP. Theoretically, the combined therapy modalities could induce a more rapid tumor response and resolution of KMP and decrease the rebound rates (15). However, we did not design a cohort study to investigate in the present report. Whether the pharmacological manipulation and ILP with percutaneous sclerotherapy could obscure the effect of each modality remains unknown. Future multicenter and large cohort research should be carried out to disclose the efficacy.

In summary, the present research presents a novel and safe multi-modality treatment for infant vascular tumors associated with KMP.

## Data Availability Statement

The raw data supporting the conclusions of this article will be made available by the authors, without undue reservation.

## Ethics Statement

The studies involving human participants were reviewed and approved by the Ethics Committee of Xinhua Hospital. Written informed consent to participate in this study was provided by the participants' legal guardian/next of kin. Written informed consent was obtained from the minor(s)' legal guardian/next of kin for the publication of any potentially identifiable images or data included in this article.

## Author Contributions

XS and MX: conception, design, and administrative support. All authors are contributed to provision of study materials or patients, collection, assembly of the data, the data analysis, interpretation, manuscript writing, and final approval of manuscript.

## Funding

This work was supported by the China National Natural Science Foundation Youth Science Fund Project (No. 81701949).

## Conflict of Interest

The authors declare that the research was conducted in the absence of any commercial or financial relationships that could be construed as a potential conflict of interest.

## Publisher's Note

All claims expressed in this article are solely those of the authors and do not necessarily represent those of their affiliated organizations, or those of the publisher, the editors and the reviewers. Any product that may be evaluated in this article, or claim that may be made by its manufacturer, is not guaranteed or endorsed by the publisher.
